# Postoperative Delirium: A Survey of Perceptions Among Surgical Providers

**DOI:** 10.56392/001c.161767

**Published:** 2026-05-21

**Authors:** Julia R. Berian, Ben L. Zarzaur, Sanjay Mohanty, Farah A. Kaiksow, Blair P. Golden

**Affiliations:** 1 Surgery, University of Wisconsin–Madison,; 2 Surgery, Indiana University School of Medicine,; 3 Medicine, University of Wisconsin–Madison

**Keywords:** Surgery, Postoperative delirium, Survey, Identification, Management, Knowledge, delirium

## Abstract

**Background:**

Postoperative delirium (POD) remains a significant, costly complication. Despite increasing awareness in medical specialties, understanding among surgical providers remains unknown.

**Objective(s):**

We aimed to (1) understand surgical providers’ self-perceived knowledge of and confidence in identifying and managing POD, and (2) define the perceived importance of POD among surgical providers, relative to other complications.

**Methods:**

An anonymous electronic survey was distributed to surgical providers across three hospitals in an academic medical system.

**Results:**

Seventy-six of 267 (28.5%) responded. The majority were somewhat (n=48, 63%) or very confident (n=12, 16%) identifying POD, however, few used validated tools (n=18, 24%). Most were somewhat (n=40, 52.6%) or very confident (n=9, 11.8%) managing POD. Few consulted geriatrics or other fields for co-management (n=23, 30.2%). Among six common complications, delirium consistently ranked third or fourth in importance.

**Conclusions:**

Future work should focus on increasing the use of validated tools and building interdisciplinary initiatives for co-management.

## INTRODUCTION

Postoperative delirium (POD) is a common complication for older adults after major surgery, associated with prolonged hospitalization, increased healthcare costs and poor health outcomes.^1–4^ The rate of POD after elective operations ranges from 10–20%, with higher rates after emergency operations (30–40%).^5–9^ By comparison, the more common “surgical” complications occur at lower rates but are easily recognized and often carry consequences for surgeons and patients. For example, surgical site infection of the incision is one of the most common complications, occurring in 3–7% of cases; it is publicly reported and has the potential to affect reimbursement rates.^10–12^ Myocardial infarction occurs less often (0.5–3%) but significantly increases risk for postoperative mortality.^10,13^

POD is often underdiagnosed or poorly managed; as a result, many efforts focus on increasing awareness of POD among healthcare professionals.^14–19^ Although it has been described as a critical knowledge gap for surgeons,^20,21^ much of the existing literature has focused on knowledge and attitudes among other healthcare professionals, including nurses, advanced practice providers, hospitalists, emergency physicians and intensive care physicians.^17–19^ Surgeons’ knowledge of and attitudes toward POD remain unexplored. Given the transient nature of POD and the range of life-threatening complications treated by surgeons, we hypothesized that surgeons may have limited knowledge of or concern for POD. We aimed to (1) understand surgical providers’ self-perceived knowledge of and confidence in identifying and managing POD, and (2) define the perceived importance of POD among surgical providers, relative to other complications.

## METHODS

We conducted an anonymous electronic survey of attending surgeons and advanced practice providers (APPs), together referred to as surgical providers, practicing across three hospitals in an academic health system. Attending surgeons and APPs each play important roles as independent providers with experience in perioperative care. In addition, some APPs deliver more direct patient-facing postoperative care than attending surgeons in our system. We revised a previously-published 14-item survey of hospitalist attitudes toward delirium, making it more relevant to surgical care.^22^ For example, we added a question to assess perceptions of POD compared to surgical complications, selected from the most common complications that are relevant in reporting surgical quality through the National Surgical Quality Improvement Program or publicly reported as required by the Centers for Medicare & Medicaid Services.^23–25^ The revised survey was pre-tested by a multidisciplinary expert panel with respondent debriefing and cognitive interviews with one surgeon and one APP.

The anonymous survey was emailed to the departmental listserv (March 2024) and remained open for two weeks. One reminder email was issued. Incomplete responses were excluded from final analysis. Responses were cleaned in Excel and analyzed in STATA with descriptive and bivariate statistical tests (chi-squared, ANOVA). The study was reviewed by the IRB and classified as exempt. No participant incentives were provided.

## RESULTS

Of 267 surgical providers, eighty-five (31.8%) responded, with 76 providing complete responses (28.5%). Most respondents were female (n=47, 61.8%), attending surgeons (n=50, 65.8%) and five or more years into practice (n=55, 72.4%). The majority of respondents (n=50, 65.8%) reported some formal training in geriatric principles, with 20 (26.3%) reporting multiple sources, including pre-clinical course-work, residency training, and CME.

Estimates of POD varied widely by specialty (0–65%, p=.0013), with no difference between surgeons and APPs. The highest estimates came from Acute Care Surgeons (including Trauma, Emergency General Surgery and Critical Care) with an average rate of 33.7% (range 10–65%), Colorectal 30% (10–50%) and Transplant 28% (10–40%). The lowest estimates came from Endocrine Surgery at 5% (0–5%) and Plastic Surgery at 2.5% (0–5%).

Overall, most respondents felt somewhat (n=48, 63.2%) or very confident (n=12, 15.8%) identifying POD. Most cases were identified by nursing (n=68, 89.5%) or “I notice it myself based on clinical gestalt” (n=63, 82.9%). Over half the respondents stated that family members helped identify cases (n=44, 57.9%). POD was infrequently identified using a validated tool (n=18, 23.7%). Half the respondents reported feeling somewhat confident (n=40, 52.6%) or very confident (n=9, 11.8%) managing delirium. Top management strategies included minimizing deliriogenic medications (n=57, 75.0%), promoting sleep-wake cycles (n=53, 69.7%), and ruling out underlying complications (n=45, 59.2%). Less than one-third of respondents consulted geriatrics (n=23, 30.3%), and very few (2, 2.6%) reported regularly using antipsychotic medications.

**Figure 1.**
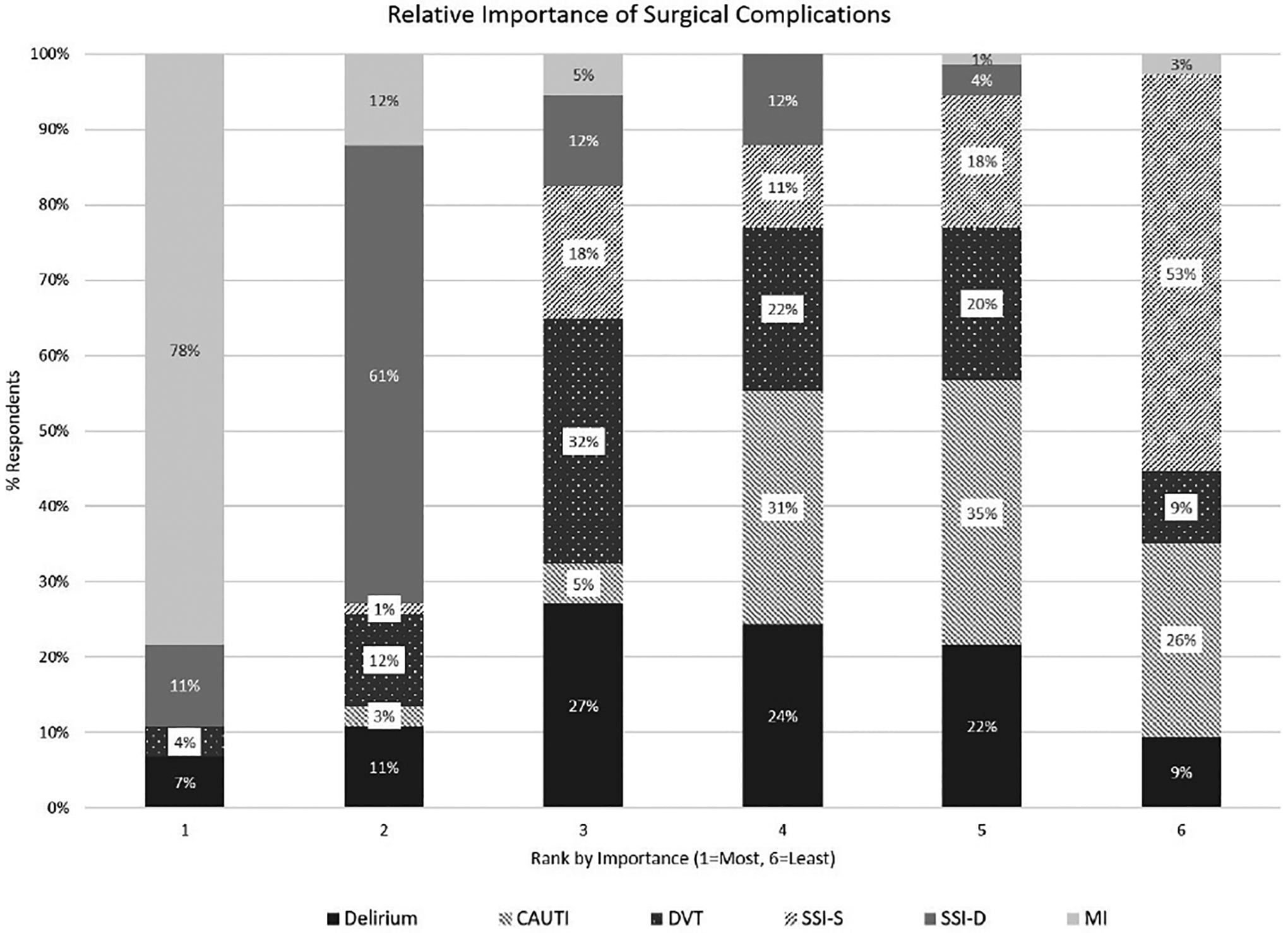
Relative Importance of Surgical Complications Respondents ranked 6 common surgical complications, including postoperative delirium in order of perceived importance. CAUTI=Catheter-Associated Urinary Tract Infection, DVT=Deep Venous Thrombosis, SSI-S=Surgical Site Infection (Superficial), SSI-D=Surgical Site Infection (Deep or Organ Space), MI=Myocardial Infarction.

**Figure 2.**
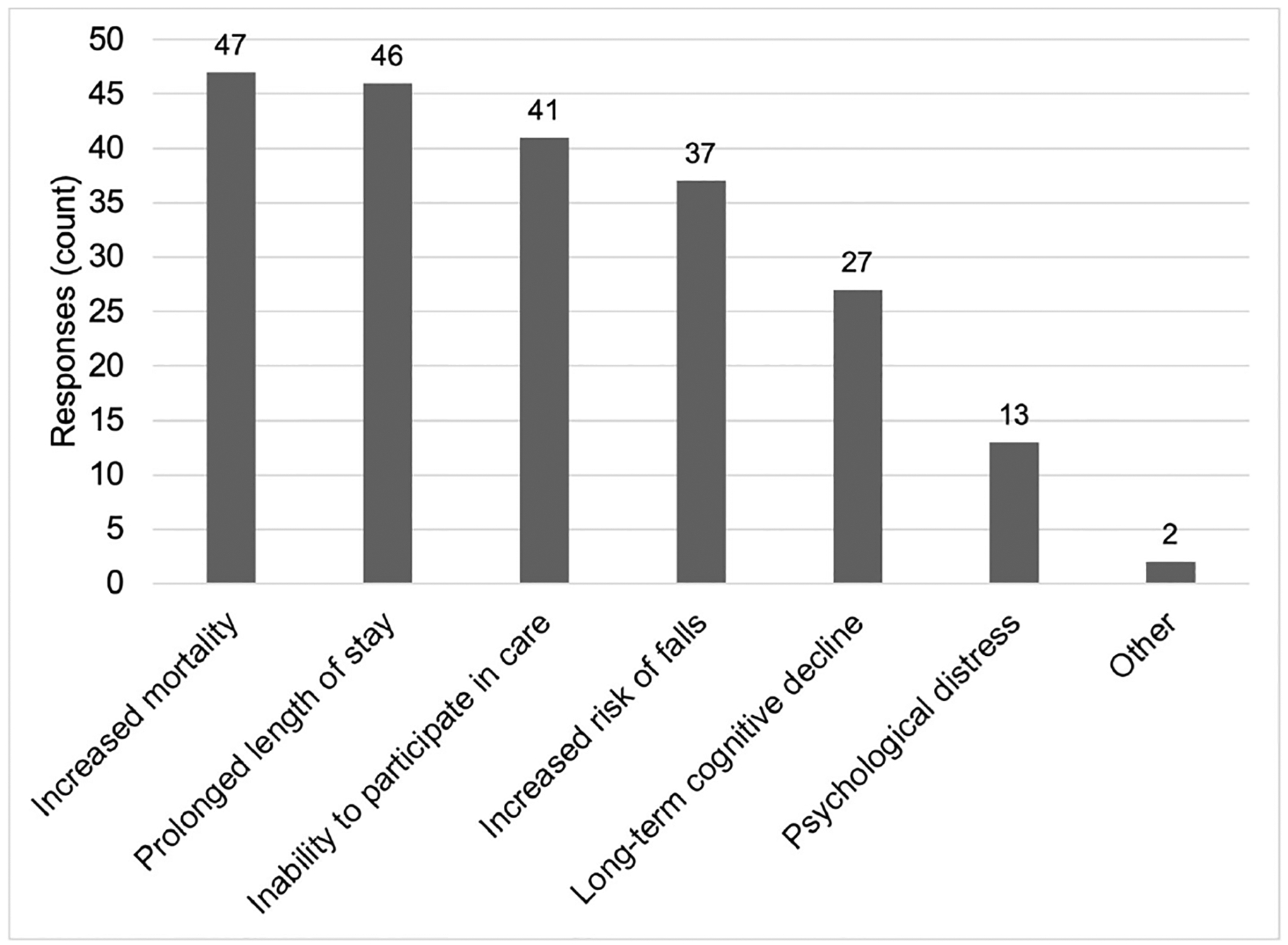
Most Concerning Consequences of Postoperative Delirium Respondents selected the 3 most concerning consequences of postoperative delirium. Other responses were added as free text and included: “loss of independence” and “confounding with other complications/diagnoses.”

Ranking six common complications in order of importance, delirium was commonly third or fourth. Cardiac complications were consistently most important, while superficial site infections ranked least important (Figure 1). Respondents recognized that POD has negative consequences (Figure 2). Some perceived the potential for persistent long-term cognitive effects (n=20, 26.3%), though most expected the effect was limited (n=44, 57.9%). Few stated delirium has no lasting effects (n=5, 6.6%) and some acknowledged they did not know (n=6, 7.9%).

Additional sensitivity analysis comparing surgeons and APPs demonstrated no statistically significant difference in strategies to identify or manage POD, however, there were significant differences with respect to confidence identifying delirium. Among the APP respondents 3/26 (11.5%) reported feeling “not confident” while among surgeons only 1/50 (2.0%) reported feeling “not confident.” More surgeons (37/50, 74.0%) than APPs (11/26, 42.3%) felt “somewhat confident” and a higher proportion of APP’s (5/26, 19.2%) than surgeons (7/50, 14.0%) felt “very confident” in identifying POD. (p=.028) There were no significant differences between surgeons and APPs with regard to confidence managing POD, once it was identified. There were no significant differences in the relative ranking of POD compared to other surgical complications or in the perceived consequences and long-term effects of POD.

## DISCUSSION

Surgeons and APPs in this study recognized POD as moderately important for older surgical patients, with implications for long-term health. Respondents report high confidence in identifying POD, but infrequently use validated tools to diagnose it. Estimated rates of POD in this study varied by specialty, with perceived higher rates in fields with a larger proportion of emergency and high-risk procedures. For example, the estimated rate of POD in Acute Care Surgery was 33.7%. By comparison, the estimated rate was 13.3% for Minimally Invasive Surgery, a field with a high proportion of elective, planned procedures. These estimates are consistent with published rates in elective vs. emergency settings.^5,6,8,26^ In this survey, POD was most often identified from nursing concerns or clinical gestalt, and less often through validated tools.27 Taken together, these findings underscore an opportunity to improve care through consistent use of validated tools.

Confidence in managing POD was low in this survey, yet a minority of respondents consulted geriatrics for co-management. These discordant findings highlight an opportunity for interdisciplinary collaboration, a recommendation from the American Geriatrics Society Clinical Practice Guideline for Postoperative Delirium in Older Adults, endorsed by multiple specialty societies.21 The majority of surgical providers reported receipt of formal training, with high self-reported confidence in understanding and identifying POD. Given these findings, educational efforts alone are unlikely to succeed in current surgical practice. Future efforts to improve POD should focus on building interdisciplinary initiatives and strategies for co-management.

Surgical care is often delivered by teams of providers, including both attending surgeons and APPs. For some surgical specialties, APPs deliver more direct patient-facing postoperative care, compared to attending surgeons. As such, some attending surgeons may not be actively involved in the identification or management of POD. This team-based care model may explain some of the findings in this study. Attendings were more likely to report feeling “somewhat comfortable” identifying POD, while APPs were more split, reporting they were either “not comfortable” or “very comfortable” identifying POD. The low rates of validated tools may reflect lack of use (e.g., low screening rate among nurses) or low awareness of their use by surgeons or APPs. While it would not be feasible for surgeons to screen for delirium themselves, these data should support the need for comprehensive interdisciplinary pathways for POD detection and management.

Limitations of this survey study include modest response rates from providers at a single institution, which may limit generalizability. There may be selection bias among respondents, with providers interested in geriatric principles preferentially responding to the survey. The survey respondents were majority female, driven in part by the predominance of female APPs in the department. These findings may not generalize well to institutions with a different distribution of male-to-female surgeons and APPs. There was no formal validation of the psychometric properties of the survey. In sum, these are preliminary data that should be replicated in larger, more diverse cohorts in future studies.

This survey adds to the literature as the first to report surgical providers’ self-reported perspectives on postoperative delirium, a common and costly complication. The findings highlight the need for ongoing efforts to track delirium with validated metrics and to develop models of care that build interdisciplinary co-management. These critical building blocks will then facilitate future research initiatives to deploy longitudinal, evidence-based interventions to prevent and treat postoperative delirium and, potentially, to mitigate its short- and long-term consequences.

## Supplementary Material

Supplement

Supplement - Postoperative Delirium Survey

Download: https://deliriumjournal.com/article/161767-postoperative-delirium-a-survey-of-perceptions-among-surgical-providers/attachment/343224.docx
