# Supplementary material for: Postoperative Delirium: A Survey of Perceptions Among Surgical Providers
Source: Delirium (Bielef). Author manuscript; Available in PMC 2026 Jul 11. (PMC13354056; doi:10.56392/001c.161767)
Supplement: Supplement [file NIHMS2185626-supplement-Supplement.docx]

(1) In your field, what percentage of patients develop postoperative delirium? (Numeric answer)

(2) What is your surgical practice?

- Acute Care Surgery (including Trauma, Burn, Surgical Critical Care)
- Cardiothoracic
- Colorectal
- Endocrine
- General Surgery
- Minimally Invasive Surgery
- Otolaryngology
- Pediatric Surgery
- Plastic Surgery
- Surgical Oncology
- Transplant Surgery
- Vascular Surgery
- Other (free text) ______________________________________________

(3) Have you received formal training on postoperative delirium (can be identification, prevention, and/or management)? (select all that apply)

- No formal training
- Pre-clinical school coursework
- Clinical rotations in medical, APP, or nursing school
- Residency training
- CME training
- Other (free text) _____________________________________________

(4) How does postoperative delirium typically come to your attention? **Select up to 3 responses.**

- I notice it myself based on clinical gestalt
- Nursing concerns
- Family member input
- Positive screen on Confusion Assessment Method (CAM) or other validated screening tool
- Geriatrics or Acute Care for Elderly (ACE) consult
- Other (free text) ___________________________________________

(5) How confident are you in your ability to identify postoperative delirium?

- Very confident
- Somewhat confident
- Neutral
- Not confident
- Other (free text) _____________________________________________

|  |
| --- |

(6) When it happens, how do you typically manage postoperative delirium? Select **up to 3 responses.** Note: If you routinely use more than 3, please select the 3 most important to you**.**

- Frequent re-orientation
- Promotion of a normal sleep wake-cycle
- Minimization of medications that exacerbate or increase the risk of delirium
- Consult another service - e.g., Geriatrics or Acute Care for Elders (ACE) teams
- Encouraging family visits
- Rule out underlying complication (e.g., infection)
- Administer anti-psychotics
- Pain control with multimodal analgesia
- Other (free text) _____________________________________________

(7) When it happens, how confident are you in your ability to manage postoperative delirium?

- Very confident
- Somewhat confident
- Neutral
- Not confident
- Other (free text) ___________________________________________

(8) In your view, how important is postoperative delirium, relative to other common postoperative complications? (Please rank from 1 most important to 6 least important)

______ Postoperative delirium

______ Catheter-associated urinary tract infection

______ Deep vein thrombosis of the lower extremity

______ Surgical site infection (superficial)

______ Surgical site infection (deep or organ space)

______ Cardiopulmonary complications (e.g., myocardial infarction)

(9) In your view, what are the most concerning consequences of delirium? Select **up to 3 responses.**

- Inability to participate in care
- Increased fall risk
- Increased length of stay
- Increased hospital costs
- Increased mortality
- Long-term cognitive impairment
- Psychologic distress from episode
- Other (free text) _____________________________________________

(10) In your experience, to what extent does postoperative delirium affect your patients' long-term health? (Specifically, this may be memory, cognitive or other delirium-related changes persisting >30 days after surgery)

- A large extent
- A limited extent
- No lasting effect
- Other (free text) ______________________________________________
